# The Effectiveness of Community-Based Continuous Training on Promoting Positive Behaviors towards Birth Preparedness, Male Involvement, and Maternal Services Utilization among Expecting Couples in Rukwa, Tanzania: A Theory of Planned Behavior Quasi-Experimental Study

**DOI:** 10.1155/2018/1293760

**Published:** 2018-09-27

**Authors:** Fabiola V. Moshi, Stephen M. Kibusi, Flora Fabian

**Affiliations:** ^1^School of Nursing and Public Health, The University of Dodoma, P.O. Box 259, Dodoma, Tanzania; ^2^School of Medicine and Dentistry, The University of Dodoma, P.O. Box 259, Dodoma, Tanzania

## Abstract

**Background:**

Rukwa Region has the highest maternal mortality ratio, 860 deaths per 100,000 live births in Tanzania. The region has neonatal mortality rate of 38 deaths per 1,000 live births. Previous interventions to promote maternal and neonatal health targeted access to maternal services by removing financial barriers and increasing the number of health facilities. However, maternal service utilization remains very low, especially facility delivery. The proposed intervention was sought to address deep-rooted behavioral beliefs, normative beliefs, control beliefs, and knowledge empowerment to determine their effect on improving birth preparedness, male involvement, and maternal services utilization. The study tested the effectiveness of a Community-Based Continuous Training (CBCT) intervention that was based upon the theory of planned behavior and was sought to promote positive behaviors.

**Methods:**

The study used a quasi-experimental design. The design consisted of pre- and postintervention assessments of two nonequivalent groups. Two districts were selected conveniently using criteria of high home birth. A district to hold intervention was picked randomly. Study participants were expecting couples at gestation age of 24 weeks and below. After obtaining informed consents, participants were subjected to baseline assessment. Expecting couples in the intervention group had two training sessions and two encounter discussions. The three primary outcomes of the study were changes in the level of knowledge about birth preparedness, male involvement, and use of maternal services. Data were collected at preintervention, midintervention, and postintervention. *Policy Implications of the Results*. The aim of this paper was to describe the study protocol of a quasi-experimental study design to test the effectiveness of an interventional program on promoting positive behaviors on birth preparedness, male involvement, and maternal services utilization among expecting couples. This study has a potential to address the challenge of low birth preparedness, male involvement, and use of maternal health services in Rukwa Region.

## 1. Background

It is estimated that 293,300 maternal deaths occurred in 2013 worldwide [1]. The major causes of these deaths were maternal hemorrhage (44,200 deaths), complications of abortion (43,700 deaths), maternal hypertensive disorders (29,300 deaths), maternal sepsis and other maternal infections (23,800 deaths), and obstructed labor (18,800 deaths) [1]. Most of these deaths occurred in the sub-Saharan Africa (62%) and South Asia (24%), which altogether account for 86% of maternal mortality worldwide [2].

The risk of a woman dying due to maternal causes in developing countries is high: one woman in every 76 deliveries [3]. Comparison of the risk in Tanzania where one woman dies in every 44 deliveries to the risk in Poland where one woman in every 22,100 deliveries dies from maternal causes was done in [3, 4]. Tanzania ranks among the countries with the highest maternal mortality rates worldwide [5]. The Tanzania's estimated maternal mortality ratio is 556/100,000 [6] meaning that, for every 1,000 live births in Tanzania, about 5 women die due to pregnancy-related causes daily, which amounts to 8,000 maternal deaths per year. The maternal mortality ratio varies within Tanzania with the highest maternal mortality of 860 deaths per 100,000 live births [7] in Rukwa Region.

Similar to maternal survival, the survival of neonates depends very much on investment in maternal care, especially access to skilled antenatal care, delivery, and early postnatal services [3]. This is because 36 per cent of all newborn deaths are due to severe infections which necessitate identification and treatment of infections during pregnancy as well as clean delivery practices [8]. Also, asphyxia (difficulty in breathing after birth) causes 23 per cent of newborn deaths and can largely be prevented by improved care during labor and delivery [8]. In spite of the fact that antenatal care, use of skilled birth attendants, and postnatal care services are important maternal health services that can significantly reduce maternal and neonatal mortalities [9], there is unacceptably low use of these services in the sub-Saharan Africa including Tanzania [10, 11].

Fifty-two percent of women in low- and middle-income countries do not receive the recommended four or more antenatal skilled care [2]. The percentage of women who attended antenatal care for four or more times increase from 37% in 1990 to 52% in 2012 worldwide [2]. However, the rate of women who attended four times or more in the sub-Saharan Africa remained as low as 44% in 2012 [2] while the average attendance in Tanzania is 51% [6].

Forty million births in developing regions were not attended by skilled health personnel, but by traditional birth attendants or a relative in 2012, and over 32 million of those births occurred in rural areas [2]. Despite the fact that the use of skilled birth attendants in developing countries was 68% in 2012, the use of skilled birth attendants in the sub-Saharan Africa was only 53% [2] and the average use of health facility generally in Tanzania in the period of 2010–2015 was 64% which is the same in Rukwa Region [6].

Studies have worked out that, two major causes of low utilization of maternal health services are low levels of birth preparedness and male involvement [12–14]. Birth preparedness has a potential to reduce all three phases of delays to access maternal services. These delays include delay in decision-making to seek health care, delay in reaching a health facility, and delay in obtaining appropriate care upon reaching a health facility [14]. Studies have reported that when male partners are involved in birth preparedness, there is an increased chance that the level of preparedness improves [13, 14]. Despite the fact that male involvement improves obstetric care-seeking behavior, the practice of male partner's involvement in developing countries is unacceptably low: 32.1% in Nigeria [15], 42.9% in Uganda [16], 18% in Burundi [17], and 12% in Tanzania [18].

Empowering men with required information about emergency obstetric conditions and engaging them in birth preparedness is a vital strategy towards improving maternal services utilization [19]. One strategy employed by the global north to reduce maternal and neonatal mortality has been to include expecting fathers in the maternal and newborn healthcare system. The most common approach in the global north, among others, is to invite men to be present at regular prenatal checkups as well as parent training [17]. The state of male involvement in these countries is as high as 80% in Denmark [20] and 90% in Sweden [21]. In the global south, male partners' presence and participation at prenatal care visits varies greatly from 96% in the Maldives [17] to only 12% in Tanzania [18], 18% in Burundi [17], and 32.1% in Nigeria [15].

Based on the impact of male partner involvement in the global north, it is now widely recognized that the same strategy should be employed in the global south to improve birth preparedness and hence maternal services utilization [17]. Also, a systematic review done in developing countries reported an improvement of maternal health outcomes when male partners are involved in pregnancy and postnatal services of their female partners [22]. It is vital that the healthcare systems in developing countries include forgotten male partners in reproductive health matters, with a particular focus on improving birth preparedness and hence maternal services utilization. The global community has now recognized this as a global priority [17].

The Tanzania health sector strategic plan 2015–2020 seeks to increase maternal services utilizations in the country. By 2020, this strategic plan seeks to increase the recommended four or more antenatal visits from 43% to 60%, institutional deliveries from 50% to 65%, deliveries assisted by skilled health attendant from 51% to 60%, and postnatal care within seven days from 56% to 68% [23]. To that end, the Community-Based Continuous Training (CBCT) was a proposed interventional program which was sought to promote birth preparedness, male involvement, and maternal services utilization among expecting couples in rural Rukwa Region, Tanzania. This is an intervention grounded in the *Theory of Planned Behavior* which tested both behavior intentions and behavior practice.

## 2. Objective

This study was sought to determine the effectiveness of community-based continuous training on promoting birth preparedness, male involvement, and maternal services utilization among expecting couples in rural Rukwa Region, Tanzania.

## 3. Methods

### 3.1. Design

**Figure 1:**
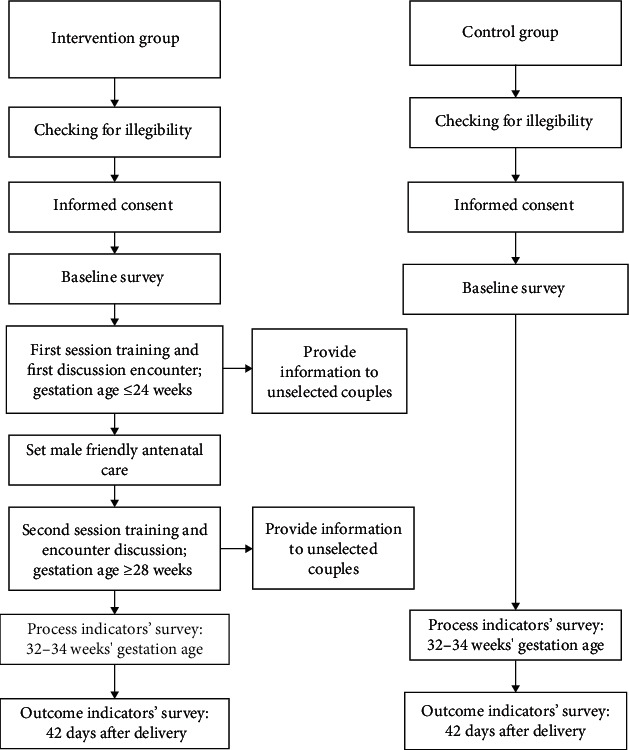
Study design flow chart.

The study was designed as a quasi-experimental study design (Figure 1). First, informed consent was obtained from all participants, followed by preintervention measures being administered.

After preintervention measures are completed, participants in the intervention group were subjected to intervention. The intervention consisted of two sessions preceded with discussions on beliefs which hinder health facility birth preparedness, male involvement, and use of available maternal services. Each individual couple was trained together but they were assessed for knowledge separately. Immediate assessment of understanding of the training was worked out from both sessions.

### 3.2. Expected Outcomes of This Study

**Figure 2:**
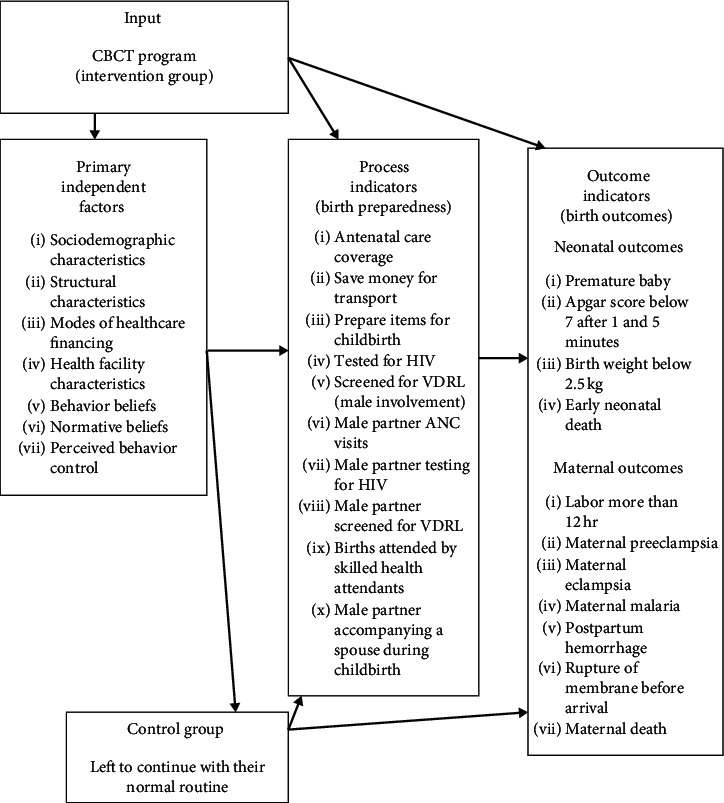
Effectiveness of Community-Based Continuous Training (CBCT) on birth preparedness and birth outcome.

The primary independent factors which influence birth preparedness, male involvement, and maternal services utilization were explored. These included sociodemographic characteristics, structural characteristics, modes of healthcare financing, health facility characteristics, behavior beliefs, normative beliefs, and perceived behavior control. Secondly, the process indicators that comprise the three key behaviors of interest were also explored. These included antenatal care coverage, saving money for transport, preparation of items for childbirth, HIV and syphilis testing, male partner ANC visits, male partner testing for HIV and syphilis, location of birth, whether a male partner accompanied the spouse during postnatal checkups or childbirth. Lastly, neonatal and maternal outcome indicators were tracked. Neonatal outcomes included gestational age, Apgar score, birth weight, and early neonatal death. Maternal outcomes included key complications of pregnancy, labor, and the postpartum period such as prolonged active labor, preeclampsia or eclampsia, maternal malaria, postpartum hemorrhage, prelabour rupture of membranes, and maternal death (Figure 2). The Directorate of Research and Publication of University of Dodoma offered ethical clearance for this study protocol.

### 3.3. Participants

Eligible study participants were expecting couples in Rukwa Region, Tanzania, who were less than 24 weeks pregnant.

### 3.4. Recruitment of Study Population

Recruitment of study participants was performed separately among the control and intervention groups. Two districts (Sumbawanga Rural District and Kalambo District) were conveniently selected from the four districts within Rukwa Region. Three staged multistage cluster sampling technique was used to obtain study participants. During first-stage random samplings, all wards in each district (12 wards of Sumbawanga Rural District and 17 wards of Kalambo District) were listed, and by the use of the lottery method of random sampling, five wards from Sumbawanga District and ten from Kalambo District were selected. During second-stage random sampling, all villages in the selected wards were listed and another simple random sampling was used to select fifteen villages from Sumbawanga Rural District and thirty villages from Kalambo District. The third-stage sampling was a systematic sampling used to obtain households with pregnant women of 24 weeks' gestation or below who were living with a male partner. At each visited household, a female partner was interviewed for the signs and symptoms of pregnancy or current known pregnancy. Any woman with amenorrhea for a minimum of two months was offered a urine pregnancy test. Those who consented were then tested during the home visit. Those with positive tests and women who already knew they were pregnant were educated about the study, and consent was sought for enrollment. Spouses of those who gave verbal and written consent to participate were consulted and a verbal and written consent was obtained from them also to participate; couples who provided consent were enrolled in the study. If a selected household had no eligible participants or refused to be tested or was tested and refused to participate in the study, the household was skipped, and the next household was visited. Recruitment of study participants into this study began in March 2017.

### 3.5. Study Inclusion and Exclusion Criteria

The inclusion criteria for this study were pregnant women 24 weeks or less who are living with their spouse. Participants were excluded if they had cognitive problems and were unable to understand or follow the training or if the spouse is traveling out of the home for work. Any woman or spouse who refused to participate was excluded. For inclusion, both the woman and the spouse had to agree to participate.

### 3.6. Sample Size

#### 3.6.1. Sample Size Calculation

The sample size for couples involved in the study was calculated using the following formula [24]:

(1) $$\begin{array}{c} n=\frac{\left\{Z\alpha \sqrt{\left[\pi 0\left(1-\pi 0\right)\right]}+2\beta \sqrt{{\left[\pi 1\left(1-\pi 1\right)\right]}^{2}}\right\}}{{\left(\pi 1-\pi 0\right)}^{2}}, \end{array}$$

where *n* = maximum sample size, *Zα* = standard normal deviation (1.96) at 95% confidence level for this study, 2*β* = standard normal deviate (0.84) with a power of demonstrating a statistically significant difference before and after the intervention between the two groups at 90%, *π*0 = proportion at preintervention (use of skilled delivery in Rukwa Region, 30.1%) [9], and *π*1 = proportion after intervention (proportion of families which will access skilled birth attendants, 51% [9]:

(2) $$\begin{array}{c} n=\frac{{\left\{1.96\sqrt{\left[0.301\left(1-0.301\right)\right]}+0.84\sqrt{\left[0.51\left(1-0.51\right)\right]}\right\}}^{2}}{{\left(0.6-0.51\right)}^{2}}, \end{array}$$

where *n* = 162 couples + 10% = 180.

Therefore, the required sample size in the intervention group is 180 couples.

*Intervention*: control ratio = 1 : 2. Therefore, sample size in the control group = 360 couples.

Parity and age in the five-year group was used as the pairing criteria.

## 4. Intervention

### 4.1. Community-Based Continuous Training (CBCT) Program

**Figure 3:**
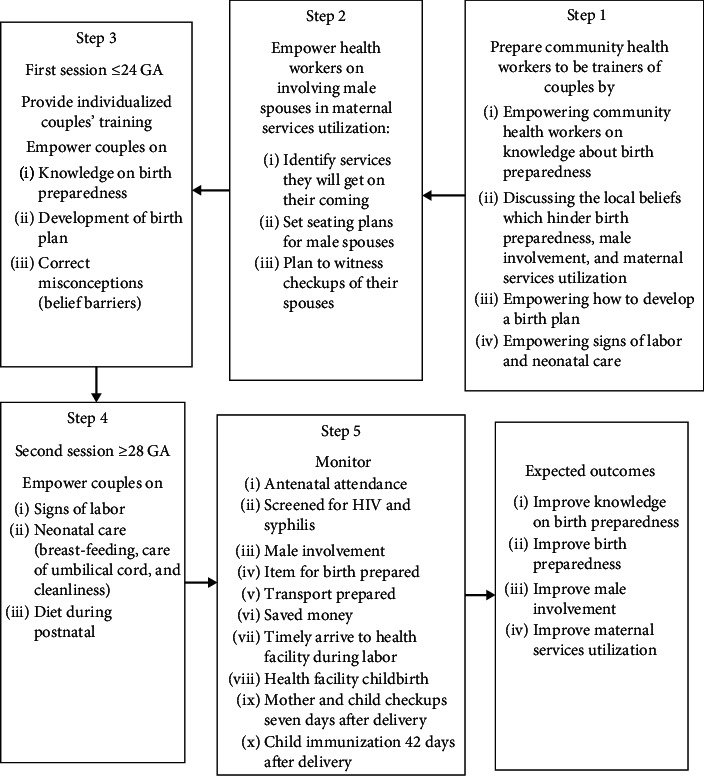
Steps involved in the interventional program.

This intervention was grounded in the *Theory of Planned Behavior.* The Community-Based Continuous Training (CBCT) program aimed to improve birth preparedness, male involvement, and maternal services utilization among expecting couples. The program involved discussions about behavior beliefs, normative beliefs, and behavior control beliefs which hinder health facility birth preparedness, male involvement, and maternal services utilization. In addition, the program involved imparting knowledge about birth preparedness (antenatal services, danger signs, and preparations for health facility birth), signs of labor, and newborn care. There were two sessions: one session about birth preparedness that was conducted prior to 24 weeks' gestation and the second session focused on signs of labor and newborn care and was conducted at 28 weeks and above. The intervention included five activities which all aimed at improving birth preparedness, male involvement, and maternal services utilization (Figure 3).

**Figure 4:**
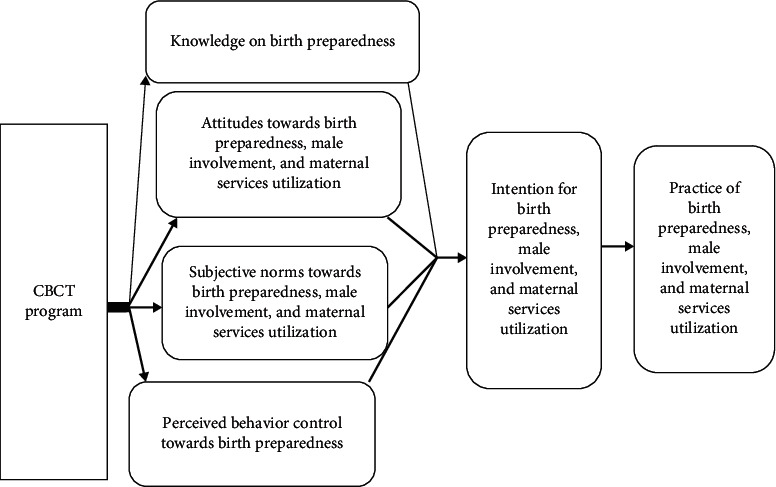
Theoretical description of the influence of the CBCT program. The thick arrows explain the influence according to the theory of planned behavior. The thin arrows represent knowledge empowerment as adding influence to the occurrence of behavior of interest.

The intervention was geared towards the domains of intention (attitudes, subjective norms, and perceived behavior control) as stated in the theory of planned behavior. The program included the fourth component, which is knowledge empowerment. They all together improve intention to the three behaviors of interest in this study, which are birth preparedness, male involvement, and maternal services utilization. According to the theory of planned behavior, when there is intention to a certain behavior, the practice of the behavior occurs (Figure 4).

### 4.2. Data Collection Procedure

Data were collected using self-administered questionnaires. Among participants with limited literacy or illiteracy, a research assistant asked questions and documented the responses. Four trained research assistants (two from each district) were recruited, trained, and participated in data collection. Questionnaires on knowledge about birth preparedness and complication readiness were adopted and modified from monitoring BPCR tools for maternal and newborn health [25]. Several studies have adopted this tool [26, 27]. After modification, it contained five sections, namely, sociodemographic information, obstetric history, danger signs (during pregnancy, labor and childbirth, 42 days after delivery, and neonatal danger signs), antenatal care, and preparations for childbirth. On knowledge on danger signs, respondents were required to recall danger signs they knew in four areas: during pregnancy, labor and childbirth, 42 days after delivery, and neonatal danger signs. Knowledge about antenatal care was measured in two areas: knowing the appropriate time for first antenatal care booking and the number of recommended antenatal visits. Knowledge about childbirth preparation was measured through recalling the preparations to be made for childbirth. Questionnaires on testing birth preparedness intention, male involvement intention, and maternal services utilization were developed using a Theory of Planned Behavior. A Likert scale was used where respondents were supposed to strongly agree, agree, neutral, strongly disagree, and disagree. There were three subparts of the statements in the Likert scale which were (i) attitudes towards birth preparedness, (ii) perceived subjective norms towards birth preparedness, and (iii) perceived behavior control towards birth preparedness.

### 4.3. Data Analysis Procedure

Descriptive statistics were used to present participation and retention rates. Means and standard deviations were presented for continuous outcome measures and frequencies, and percentages will be presented for categorical variables.

The preintervention characteristics of participants in each condition (intervention and waitlist) will be compared. Chi-squared tests will be used to compare any group differences for categorical variables, and *t*-tests will be used for continuous variables. Missing data will be explored following data collection, and depending on the amount and type of missing data, a range of methods may be used. It is likely that imputation techniques will be used to extrapolate missing data.

Univariate and multivariate analyses will be conducted to examine the effects of community-based continuous training in the intervention group in comparison with the control group. All analyses will use an intent-to-treat approach using data from all participants who completed preintervention measures. In addition to intent-to-treat analyses, per protocol analyses will be conducted using data from participants who attended two sessions of the intervention and who completed measures at more than one time point. SPSS (Statistical Package for the Social Sciences) will be used to conduct the analyses, and estimates of effects will be calculated and reported.

### 4.4. Time Frame for the Study

The total duration of this study is 24–28 months. First, ethical approval and preparatory work took approximately 6 months. Recruitment started from March 2017 and lasted 10 months. Follow-up lasted 4 months. We anticipated that it will take between 3 and 6 months to analyze the data and report the findings. The duration of participation in the study for participants is approximately 30–36 weeks.

## 5. Discussion

The aim of this paper was to describe the study protocol of a community-based continuous training program which was sought to promote birth preparedness, male involvement, and maternal services utilization among expecting couples in rural Tanzania. Expecting couples in the intervention group were subjected to two training sessions and discussion of beliefs which hinder birth preparedness, male involvement, and maternal services utilization.

In the literature, there have been many interventions which seek to address the issue of maternal mortality. Some interventions have focused on improving birth preparedness and birth outcomes. A community-based education program in Pakistan which was called Information and Education for Empowerment and Change (IEEC) was an intervention geared towards reduction of maternal mortality. The program trained women and their husbands on the identification of obstetric danger signs. The intervention increased the utilization of health facilities for prenatal services and during obstetric complications [28]. The intervention revealed the power of involving male partners on important messages of maternal health.

Another intervention in Eritrea was a community intervention for promotion of safe motherhood. This program focused on training to improve knowledge, attitudes, and practices of birth preparedness. The program trained 30 maternal health volunteers who then lead participatory educational sessions on safe motherhood topics with women and men in the community. It reported a significant increase in knowledge about obstetric danger signs in the intervention group compared to the comparison group. The rate of antenatal attendance also showed a significant increase compared to the comparison group. The proportion of women who delivered in a health facility increased from 3% to 47% in the intervention group compared to an increase of 4% to 15% in the comparison group [29].

The *Wazazi na Mwana* project in Rukwa Tanzania is another intervention program which focused on improving maternal and neonatal health outcomes. The project had the objective of reducing child mortality and improving maternal health through community-based maternal, newborn, and child health services. The project improved couple communication and shared decision-making [30].

Involvement of husbands in antenatal training sessions was tried in Nepal, where women who were receiving antenatal services were arranged into three groups: women who received education with their husband, women who received education alone, and women who were left to continue with routine. The findings indicated that women who received education with their husband were almost twice as prepared for birth compared to women who received education alone [31]. Another study which was conducted in Nepal by Mullany et al. [32] reported an increase in knowledge among women who were trained with their husband compared with those trained alone.

Furthermore, a community-based training about safe motherhood was implemented in southern Tanzania. The key element of the intervention was the training of safe motherhood promoters using participatory adult learning methods. They conducted home visits to educate pregnant women, their husbands, and key community members about common danger signs. These home visits also sought to encourage early booking for ANC services and to review the recommended antenatal visits and birth preparedness steps. The health volunteers encouraged skilled birth delivery and raised awareness about maternal health issues through community meetings and video shows. The study reported a significant increase in deliveries assisted by skilled birth attendants (from 34.1% to 51.4%), a significant increase in early antenatal booking (from 18.7% to 56.9%), and an increase in male involvement [33].

The project aims at building the bridge between the community and healthcare system. Expecting couples were empowered on the obstetric danger signs, what to prepare for childbirth, and the antenatal services offered at their local health facilities. The primary goal was to fill the existing gap between the community and the local health facility by inducing a demand [34].

By conducting this study, we hope to add to the literature as follows: first, the involvement of male partners in birth preparedness and the measurement of the effect of their involvement on improving health facility birth preparedness and maternal services utilization. The study is unique as it used behavior theory to impart positive behavior towards birth preparedness, male involvement, and maternal services utilization. It is an experimental study which is geared to establish the effectiveness of intervention towards birth preparedness, male involvement, and maternal services utilization.

## 6. Conclusion

The key strength of this study protocol is the originality of the research. The proposed intervention has a potential to reduce the three delays which are delay in decision-making to seek health care, delay in reaching a health facility, and delay in obtaining appropriate care upon reaching a health facility. There were some limitations with this study. The study was a quasi-experimental study that lacked random assignment of study participants into either intervention or control group. This limitation was minimized by using the probability sampling technique in obtaining study respondents. Exclusion of expecting mothers who are not living with their spouse is another limitation of this study protocol. The unselected expecting couples and the pregnant women who were not living with their male spouses were given reading material in the intervention district.

## Data Availability

The data used to support the findings of this study are available from the corresponding author upon request.

## Abbreviations

ANC: Antenatal clinic
BPP: Birth preparedness plan
CBCT: Community-Based Continuous Training
CRP: Complication readiness plan
FANC: Focused antenatal care
GA: Gestation age
HIV: Human immunodeficiency virus
HPM: Health promotion model
IEEC: Information and Education for Empowerment and Change
IPT: Intermittent preventive treatment
Jhpiego: Johns Hopkins Program for International Education in Gynecology and Obstetrics
MDG: Millennium development goals
MoHSW: Ministry of Health and Social Welfare
NBS: National Bureau of Statistics
PI: Plan international
SP: Sulfadoxine and pyrimethamine
STI: Sexually transmitted infections
SPSS: Statistical Package for the Social Sciences
TPB: Theory of planned behavior
TRA: Theory of reasoned action
TT: Tetanus toxoid
UN: United Nations
UNICEF: United Nations International Children's Emergency Fund
VHW: Village health workers
VDRL: Venereal Disease Research Laboratory
WHO: World Health Organization.

## Supplementary Materials

Supplementary Materials Supplementary materials contain first questionnaire (female partners) and second questionnaire (male partners).
