# Supplementary material for: The Effectiveness of Community-Based Continuous Training on Promoting Positive Behaviors towards Birth Preparedness, Male Involvement, and Maternal Services Utilization among Expecting Couples in Rukwa, Tanzania: A Theory of Planned Behavior Quasi-Experimental Study
Source: J Environ Public Health. 2018 Sep 27;2018:1293760. doi: 10.1155/2018/1293760 (PMC6180969; doi:10.1155/2018/1293760)
Supplement: Supplementary Materials — contain first questionnaire (female partners) and second questionnaire (male partners). [file 1293760.f1.pdf]

APPENDIX: QUESTIONNAIRES

First questionnaire: Female Partners

Part A: Baseline Information

Please tick (√) the appropriate option

1. Age in years in years______________
2. Age at marriage in years_________
3. Marital status
   a) Married ( )
   b) Cohabited ( )
4. Educational status
   a) None ( )
   b) Primary level incomplete ( )
   c) Primary level complete ( )
   d) Secondary or higher ( )
5. Employment status
   a) Employed ( )
   b) Unemployed ( )
6. Religion
   a) Christian ( )
   b) Muslims ( )
   c) Others specify………………………
7. Ethnic group
   a) Fipa ( )
   b) Mambwe ( )
   c) Others specify………………………
8. Economic status
   a) Use less than one dollar per day ( )
   b) Use more than one dollar per day ( )
9. Do you own radio?
   a) Yes ( )
   b) No ( )
10. Do you own mobile phone?
    a) Yes ( )
    b) No ( )
11. Characteristic of a nearby health facility
    a) Dispensary ( )
    b) Health center ( )
    c) Hospital ( )
12. What is the walking distance to a nearby health facility?
    a) Less than one kilometer ( )
    b) One kilometer-5kilometers ( )
    c) More than five kilometers ( )

13. Are you covered with health Insurance (NHIF or CHF)?
    a) Yes ( )
    b) No ( )

14. Pregnancy history
    a) Primigravida ( )
    b) Para 1-Para 4 ( )
    c) Para 5+ ( )

15. Age at 1st delivery (years)____________

16. Current pregnancy characteristics
    a) Planned ( )
    b) Unplanned ( )

17. Did you have prior pre-term delivery?
    a) Yes ( )
    b) No ( )
    c) Not applicable ( )

18. Did you have a prior C-section?
    a) Yes ( ) b) No ( ) c) Not applicable ( )

19. Have you ever heard the term "birth preparedness"?
    a) Yes ( ) b) No ( )

20. Where did you hear about birth preparedness?
    a) From health worker ( )
    b) From the media ( )
    c) From a family member ( )
    d) Others specify____

Part B: Knowledge on Birth Preparedness

Now I would like to ask you some questions about pregnancy and childbirth. Specifically, I am going to be asking you questions about three different phases that women go through when having a child. These phases are the period of being pregnant, the period of labor and birth, and the period immediately after the birth of the child (JHPIEGO, 2004).

21. Can unforeseen problems related to pregnancy occur during any pregnancy or childbirth that could endanger the life of a woman?
    a) Yes ( ) b) No ( )

22. What are some serious health problems that can occur during pregnancy that could endanger the life of a pregnant woman?

a) ______________ d) __________________

b) ______________ e)____________________

c) _______________ f) ___________________

23. Could a woman die from [this problem] any of these problems?

a) Yes ( ) b) No ( )

24. What are some serious health problems that can occur during labor and childbirth that could endanger the life of a pregnant woman?

a) ______________ d) __________________

b) ______________ e)____________________

c) _______________ f) ___________________

25. Could a woman die from [this problem] any of these problems?

a) Yes ( ) b) No ( )

26. What are some serious health problems that can occur during the first 42 days after birth that could endanger the life of the woman?

a) ______________ d) __________________

b) ______________ e)____________________

c) _______________ f) ___________________

27. Could a woman die from [this problem] any of these problems?

a) Yes ( ) b) No ( )

28. Now, I would like to ask you a few questions about the health of newborn babies. What are some serious health problems that can occur during the first 7 days after birth that could endanger the life of a newborn baby?

a) ______________ d) __________________

b) ______________ e)____________________

c) _______________ f) ___________________

29. Could a newborn baby die from [this problem] any of these problems?

a) Yes ( ) b) No ( )

30. What are the six key elements of birth preparedness?

a) ______________ d) __________________

b) ______________ e)____________________

c) _______________ f) ___________________

31. When a pregnant woman has to start antenatal clinic?

a) Below 16 weeks gestation age ( )
b) 17-24 weeks ( )
c) Above 24 weeks ( )

32. What are the recommended antenatal visits

a) Four or more ( )
b) Three ( )
c) Two ( )
d) One ( )

Part C: Female partners behavior intentions on birth preparedness, male involvement and maternal services utilization

Many questions in this survey make use of rating scales with 4 places; you are to tick (√) the box that best describes your opinion where SA= strongly agree, A= agree, N= Neutral, D= disagree and SD = strongly disagree

Component	SA(5)	A (4)	N (3)	D (2)	SD(1)
**A: Female partners behavior intentions on birth preparedness**					
Assessment of attitude					
1. If I make arrangement for transport to be used in case emergency, I am doing something good.					
2. If I mobilize resources to assist during childbirth, I am doing a positive thing.					
3. If I identify skilled birth attendant during pregnancy, I am doing a positive thing.					
4. If I identify a relative who will donate a blood in case of emergency I am doing something good					
5. Preparation for birth ensure timely access to skilled birth attendant					
Assessment of subjective norms					
6. Important people to me think I should prepare transport during pregnancy					
7. Important people to me think I should mobilize resources during pregnancy					
8. Important people to me think I should identify skilled birth attendants during pregnancy.					
9. Important people to me think I should identify a relative who will donate blood in case of emergency.					

10. When it comes to preparation for birth I will do what the health providers advice me to do.					
Perceived Behavioral Control					
11. For me to prepare transport during pregnancy is trouble-free and I can do it					
12. For me to mobilize resources during pregnancy to be used during childbirth is effortless and I can do it					
13. For me to identify skilled birth attendants during pregnancy is simple and I can do it					
14. For me to identify a relative who will donate blood in case of emergency is simple and I can do it.					
15. Preparation for birth is within my control					
**B: Female partners' behavior intentions to utilize available maternal services**					
Attitude					
16. If I attend antenatal clinic four or more times, I am doing a good thing					
17. If I get vaccinated against tetanus toxoid, I am doing a good thing					
18. If I test for HIV during antenatal visits, I am doing a good thing					
19. If I test for Syphilis during pregnancy, I am doing a good thing					
20. If I attend for skilled birth attendant, I am doing a good thing					
21. If I attend for skilled postnatal service seven days after delivery, I am doing a good thing					
22. When I utilize available maternal services, I will ensure good birth outcome					
Assessment of subjective norms					

23. Important people to me think I should attend four or more antenatal visits					
24. Important people to me think I should get vaccinated against tetanus					
25. Important people to me think I should test for HIV during pregnancy					
26. Important people to me think I should get testes for syphilis during pregnancy					
27. Important people to me think I should use skilled birth attendants during childbirth					
28. Important people to me think I should attend postnatal care seven days after delivery					
29. When it comes to maternal services utilization, I will do what the health care provider advice me to do					
Perceived Behavioral Control					
30. For me to attend four or more antenatal clinics is simple and I can do it					
31. For me to get vaccinated against tetanus is simple and I can do it					
32. For me to be tested for HIV is trouble free and I can do it					
33. For me to be screened for STI such as syphilis is trouble free and I can do it					
34. For me to use skilled services for delivery is simple and I can do it					
35. For me to attend for postnatal checkups after seven days delivery is trouble free and I can do it					
36. For me to use available maternal health services is simple and I can do so.					
**C: Male involvement**					

Attitude					
37. If my husband participate in setting aside some funds and equipments which will be used in case of emergency or during delivery is doing a good thing which is beneficial					
38. If my husband participate in preparation of transport which will be used in case of emergency or during childbirth is doing a good which is beneficial					
39. If my husband participate in identification of skilled attendant is doing a good thing which is beneficial					
40. If my husband accompany me during antenatal clinics is doing a good thing which is beneficial					
41. If my husband test for HIV with me during pregnancy is doing a good thing which is beneficial					
42. If my husband test for syphilis with me during antenatal clinics he is doing a good thing which is beneficial					
43. If my husband accompany me during childbirth he is doing a good thing which is beneficial					
44. If my husband accompany me for postnatal checkups he is doing a good thing which is beneficial					
Assessment of subjective norms					
45. Important people to me think my husband has to participate in setting aside funds and equipments to be used in case of emergency or during childbirth.					
46. Important people to me think my husband has participate in preparation of transport to be used in case of emergency or during childbirth					
47. Important people to me think my husband should participate in identification of skilled birth attendant					

48. Important people to me think my husband has to accompany me during antenatal clinics					
49. Important people to me think my husband has to test for HIV with me during antenatal visits					
50. Important people to me think my husband has to screen for syphilis with me during antenatal visit					
51. Important people to me think my husband has to accompany me during childbirth					
52. Important people to me think my husband has to accompany me during postnatal checkups					
Perceived Behavioral Control					
53. For my husband to participate in setting aside funds and equipments to be used in case of emergency or during childbirth is trouble free and he can do it					
54. For my husband to participate in preparation of transport to be used in case of emergency or during childbirth is simple and he can do it					
55. For my husband to participate in identification of skilled birth attendant is trouble free and he can do it					
56. For my husband to accompany me during antenatal clinics is simple and he can do it					
57. For my husband to test for HIV/AIDS with me during antenatal visits is trouble free and he can do it					
58. For my husband to test for syphilis with me during antenatal clinics is simple and he can do it					
59. For my husband to accompany me during labor and childbirth is simple and he can do it.					
60. For my husband to accompany me during postnatal checkup is trouble free and he can do it					

Second questionnaire: Male Partners

Part A: Baseline Information

Please tick (√) the appropriate option

1. Age in years in years______________
2. Age at marriage in years_________
3. Phone number__________________
4. Marital status
   a) Married ( )
   b) Cohabited ( )
5. Educational status
   a) None ( )
   b) Primary level incomplete ( )
   c) Primary level complete ( )
   d) Secondary or higher ( )
6. Employment status
   a) Employed ( )
   b) Unemployed ( )
7. Religion
   a) Christian ( )
   b) Muslims ( )
   c) Others specify…………………
8. Ethnic group
   a) Fipa ( )
   b) Mambwe ( )
   c) Others specify………………
9. Economic status
   a) Use less than one dollar per day ( )
   b) Use more than one dollar per day ( )
10. Do you own radio?
    a) Yes ( )
    b) No ( )
11. Do you own mobile phone?
    a) Yes ( )
    b) No ( )
12. Characteristic of a nearby health facility
    a) Dispensary ( )
    b) Health center ( )
    c) Hospital ( )
13. What is the walking distance to a nearby health facility?
    a) Less than one kilometer ( )
    b)One kilometer-5kilometers ( )
    c)More than five kilometers ( )
14. Are you covered with health Insurance (NHIF or CHF)?
    a) Yes ( )
    b) No ( )

15. Have you ever heard the term "birth preparedness"?

a) Yes ( ) b) No ( )

16. Where did you hear about birth preparedness?

a) From health worker ( )

b) From the media ( )

c) From a family member ( )

d) Others specify____

Part B: Knowledge on Birth Preparedness

Now I would like to ask you some questions about pregnancy and childbirth. Specifically, I am going to be asking you questions about three different phases that women go through when having a child. These phases are the period of being pregnant, the period of labor and birth, and the period immediately after the birth of the child (JHPIEGO, 2004).

17. In your opinion, can unforeseen problems related to pregnancy occur during any pregnancy or childbirth that could endanger the life of a woman?

a) Yes ( ) b) No ( )

18. What are some serious health problems that can occur during pregnancy that could endanger the life of a pregnant woman?

a) ______________ d) __________________

b) ______________ e)____________________

c) _______________ f) ___________________

19. In your opinion, could a woman die from [this problem] any of these problems?

b) Yes ( ) b) No ( )

20. What are some serious health problems that can occur during labor and childbirth that could endanger the life of a pregnant woman?

a) ________________ d)______________________

b) ________________ e)_______________________

c) _______________ f)__________________

21. In your opinion, could a woman die from [this problem] any of these problems?

a) Yes ( ) b) No ( )

22. What are some serious health problems that can occur during the first 42 days after birth that could endanger the life of the woman?

a) ______________ d) __________________

b) ______________ e)____________________

c) _______________ f) ___________________

23. In your opinion, could a woman die from [this problem] any of these problems?

a) Yes ( ) b) No ( )

24. Now, I would like to ask you a few questions about the health of newborn babies. What are some serious health problems that can occur during the first 7 days after birth that could endanger the life of a newborn baby?

a) ______________ d) __________________

b) ______________ e)____________________

c) _______________ f) ___________________

25. In your opinion, could a newborn baby die from [this problem] any of these problems?

a) Yes ( ) b) No ( )

26. What are some the six key elements of birth preparedness?

a) ______________ d) __________________

b) ______________ e)____________________

c) _______________ f) ___________________

27. When a pregnant woman has to start antenatal clinic?

a) Below 16 weeks gestation age ( )

b) 17-24 weeks ( )

c) Above 24 weeks ( )

28. What are the recommended antenatal visits

a) Four or more ( )

b) Three ( )

c) Two ( )

d) One ( )

e)

Part C: Male partners behavior intentions to birth preparedness and maternal services utilization

Many questions in this survey make use of rating scales with 5 places; you are to tick (√) the box that best describes your opinion where SA= strongly agree, A= agree, N= Neutral, D= disagree and SD = strongly disagree

Component	SA(5)	A (4)	N (3)	D (2)	SD(1)
**A: Male partners behavior intentions on birth preparedness**					
Assessment of attitude					
1. If I make arrangement for transport to be used in case emergency, I am doing something good.					
2. If I mobilize resources to assist during childbirth, I am doing a positive thing.					
3. If I identify skilled birth attendant during pregnancy, I am doing a positive thing.					
4. If I identify a relative who will donate a blood in case of emergency I am doing something good					
5. Preparation for birth ensure timely access to skilled birth attendant					
Assessment of subjective norms					

6. Important people to me think I should prepare transport during pregnancy					
7. Important people to me think I should mobilize resources during pregnancy					
8. Important people to me think I should identify skilled birth attendants during pregnancy.					
9. Important people to me think I should identify a relative who will donate blood in case of emergency.					
10. When it comes to preparation for birth I will do what the health providers advice me to do.					
Perceived Behavioral Control					
11. For me to prepare transport during pregnancy is trouble-free and I can do it					
12. For me to mobilize resources during pregnancy to be used during childbirth is effortless and I can do it					
13. For me to identify skilled birth attendants during pregnancy is simple and I can do it					
14. For me to identify a relative who will donate blood in case of emergency is simple and I can do it.					
15. Preparation for birth is within my control					
**B: Female partners' behavior intentions to utilize available maternal services**					
Attitude towards birth preparedness					
16. If my wife attend antenatal clinic four or more times, she is doing a good thing					
17. If she gets vaccinated against tetanus toxoid, she is doing a good thing					

18. If she tests for HIV during antenatal visits, she is doing a good thing					
19. If she tests for Syphilis during pregnancy, she is doing a good thing					
20. If she attends for skilled birth attendant, she is doing a good thing					
21. If she attends for skilled postnatal service seven days after delivery, she is doing a good thing					
22. If she utilizes available maternal services, she will ensure good birth outcome					
Assessment of subjective norms					
23. Important people to me think my wife should attend four or more antenatal visits					
24. Important people to me think she should get vaccinated against tetanus					
25. Important people to me think she should test for HIV during pregnancy					
26. Important people to me think she should get testes for syphilis during pregnancy					
27. Important people to me think she should use skilled birth attendants during childbirth					
28. Important people to me think she should attend postnatal care seven days after delivery					
29. When it comes to maternal services utilization, she will do what the health care provider advice me to do					
Perceived Behavioral Control					
30. For my wife to attend four or more antenatal clinics is simple and she can do it					
31. For my wife to get vaccinated against tetanus is simple and she can do it					

32. For my wife to be tested for HIV is trouble free and she can do it					
33. For my wife to be screened for STI such as syphilis is trouble free and she can do it					
34. For my wife to use skilled services for delivery is simple and I can do it					
35. For my wife to attend for postnatal checkups after seven days of delivery is trouble free and she can do it					
36. For my wife to use available maternal health services is simple and she can do so.					
**C: Male involvement**					
Attitude towards male involvement					
37. If I participate in setting aside some funds and equipments which will be used in case of emergency or during delivery I am doing a good thing					
38. If I participate in preparation of transport which will be used in case of emergency or during childbirth I am doing a good thing					
39. If I participate in identification of skilled attendant I am doing a good thing					
40. If I accompany my wife during antenatal clinics I am doing a good thing					
41. If I test for HIV with my wife during pregnancy I am doing a good thing					
42. If I test for syphilis with my wife during antenatal clinics I am doing a good thing					
43. If I accompany my wife during childbirth I am doing a good thing					

44. If I accompany my wife for postnatal checkups I am doing a good thing which is beneficial					
Assessment of subjective norms					
45. Important people to me think I have to participate in setting aside funds and equipments to be used in case of emergency or during childbirth.					
46. Important people to me think I have to participate in preparation of transport to be used in case of emergency or during childbirth					
47. Important people to me think I should participate in identification of skilled birth attendant					
48. Important people to me think I have to accompany my wife during antenatal clinics					
49. Important people to me think I have to test for HIV with my wife during antenatal visits					
50. Important people to me think I have to screen for syphilis with my wife during antenatal visit					
51. Important people to me think I have to accompany my wife during childbirth					
52. Important people to me think I have to accompany my wife during postnatal checkups					
Perceived Behavioral Control					
53. For me to participate in setting aside funds and equipments to be used in case of emergency or during childbirth is trouble free and I can do it					
54. For me to participate in preparation of transport to be used in case of emergency or during childbirth is simple and I can do it					

55. For me to participate in identification of skilled birth attendant is trouble free and I can do it					
56. For me to accompany my wife during antenatal clinics is simple and I can do it					
57. For me to test for HIV/AIDS with my wife during antenatal visits is trouble free and I can do it					
58. For me to test for syphilis with my wife during antenatal clinics is simple and I can do it					
59. For me to accompany my wife during labor and childbirth is simple and I can do it.					
60. For me to accompany my wife during postnatal checkup is trouble free and I can do it					
